# Retraction Note: Efficacy and safety of atypical antipsychotic drug treatment for dementia: a systematic review and meta-analysis

**DOI:** 10.1186/s13195-016-0197-7

**Published:** 2016-07-07

**Authors:** Lin Tan, Lan Tan, Hui-Fu Wang, Jun Wang, Chen-Chen Tan, Meng-Shan Tan, Xiang-Fei Meng, Chong Wang, Jin-Tai Yu

**Affiliations:** College of Medicine and Pharmaceutics, Ocean University of China, Qingdao, 266000 China; Department of Neurology, Qingdao Municipal Hospital, School of Medicine, Qingdao University, Qingdao, 266071 China; Department of Neurology, Qingdao Municipal Hospital, Nanjing Medical University, Nanjing, 210000 China

## Retraction note

This article has been retracted by the authors after discovering an error in the data. The two CATIE-AD articles included in the systematic review [1, 2] should not have been treated as separate trials as this led to data from some trial participants being included twice in the meta-analysis. As a result, large sections of the article are inaccurate. To avoid confusion this article has been retracted and the authors have been given the opportunity to resubmit their corrected data.

[1] Schneider LS, Tariot PN, Dagerman KS, Davis SM, Hsiao JK, Ismail MS, et al. Effectiveness of atypical antipsychotic drugs in patients with Alzheimer’s disease. N Engl J Med. 2006;355:1525-38.

[2] Sultzer DL, Davis SM, Tariot PN, Dagerman KS, Lebowitz BD, Lyketsos CG, et al. Clinical symptom responses to atypical antipsychotic medications in Alzheimer’s disease: phase 1 outcomes from the CATIE-AD effectiveness trial. Am J Psychiatry. 2008;165:844-54.
